# Supplementary material for: Bulk valley transport and Berry curvature spreading at the edge of flat bands
Source: Nat Commun. 2020 Nov 3;11:5548. doi: 10.1038/s41467-020-19284-w (PMC7641251; doi:10.1038/s41467-020-19284-w)
Supplement: Supplementary file 1 — Supplementary Information [file 41467_2020_19284_MOESM1_ESM.pdf]

SUPPLEMENTARY INFORMATION

Bulk valley transport and Berry curvature spreading at the edge of flat bands

Subhajit Sinha[1†], Pratap Chandra Adak[1†*], R.S. Surya Kanthi[1], Bheema Lingam Chittari[2], L. D. Varma Sangani[1], Kenji Watanabe[3], Takashi Taniguchi[3], Jeil Jung[2], and Mandar M. Deshmukh[1*]

[1]*Department of Condensed Matter Physics and Materials Science, Tata Institute of Fundamental Research, Mumbai 400005, India*

[2]*Department of Physics, University of Seoul, Seoul 02504, Korea*

[3]*National Institute for Materials Science, 1-1 Namiki, Tsukuba 305-0044, Japan*

[†]These authors contributed equally: Subhajit Sinha, Pratap Chandra Adak

[*]pratapchandraadak@gmail.com (PCA), deshmukh@tifr.res.in (MMD)

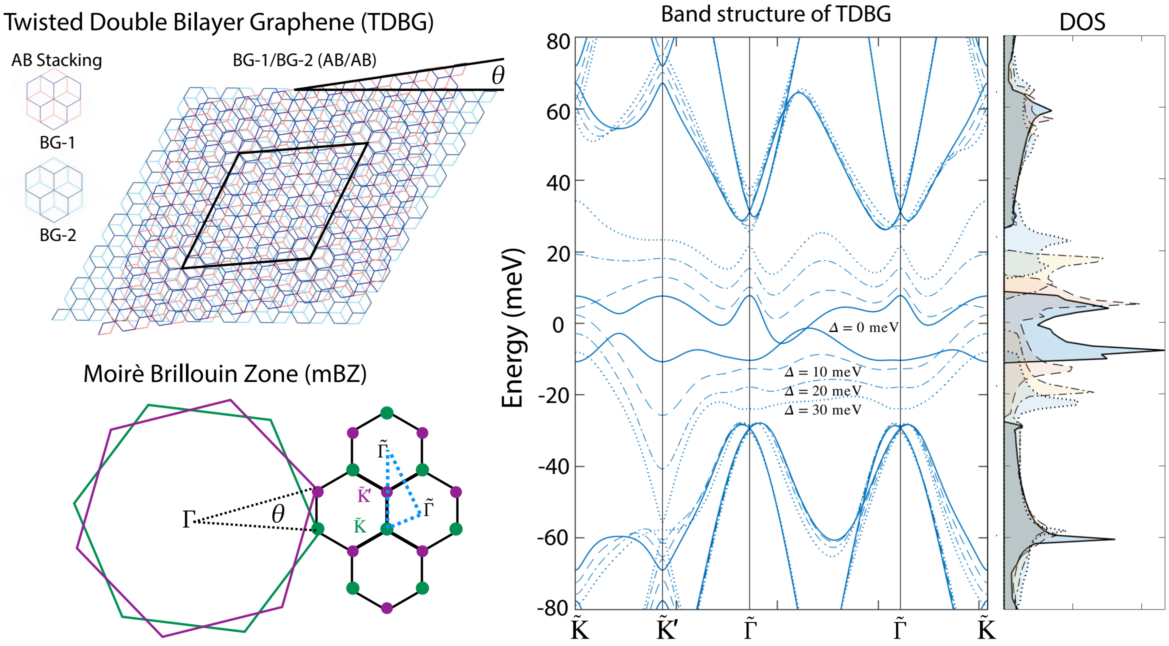

Supplementary Figure 1: Band structure of TDBG. The twisted double bilayer graphene (TDBG) is the manifestation of two bilayer graphene (BG, i.e., BG-1 and BG-2) with AB stacking on top of each other like BG-1/BG-2 (AB/AB) and rotated along the vertical axis (perpendicular to the plane) with $\theta \neq 0$. The primitive cell of moiré cell is represented with solid lines of a hexagonal unit cell. The resulting moiré Brillouin zone (mBZ) is formed from the relative displacement of K-points of each bilayer graphene unrotated Brillouin zones. The high symmetry points of mBZ are represented with a tilde to differentiate from the unrotated BZ. We calculated the band structure along the high symmetry points applied interlayer potential difference (Δ) at a twist angle $\theta = 1.18°$. The solid lines are for $\Delta = 0$ meV, dashed lines for $\Delta = 10$ meV, dashed-dot lines for $\Delta = 20$ meV and finally, the dotted lines for $\Delta = 30$ meV. The increase in the Δ separates the low energy bands near to the charge neutrality point (CNP) opening a primary gap (δ_p) and pushes these bands towards the higher energy bands into the moiré gaps between the low energy bands and higher energy bands (secondary gaps, δ_s); eventually these moiré gaps get close for large values of Δ. We also represented the density of states (DOS) obtained for different Δ values, and as represented in the band structures, the different lines in the DOS represent each Δ, as mentioned earlier. For the higher values of Δ, the moiré gaps get closed, with a primary gap opening.

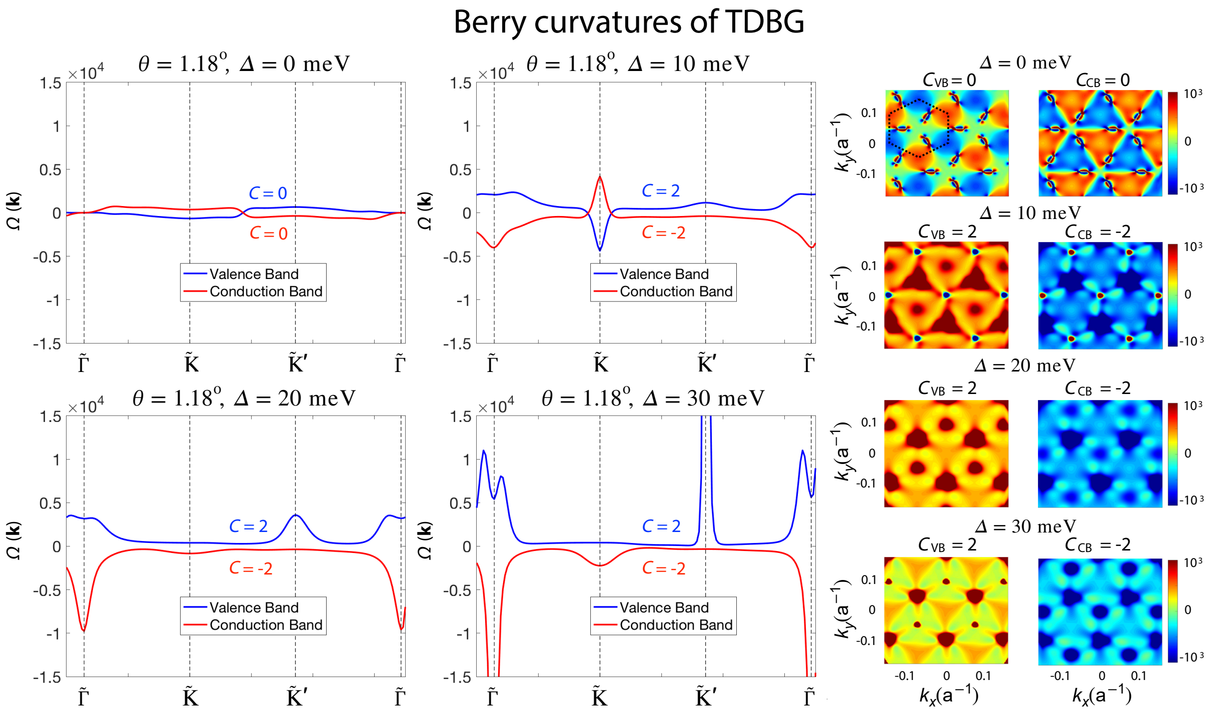

Supplementary Figure 2: Berry curvatures of TDBG. We calculate the Berry curvatures (Ω) for TDBG at a twist angle $\theta = 1.18^{\circ}$ for the different applied interlayer potential (Δ). On the left side, we represent the Berry curvature lines along with the high symmetry points for the different values of Δ. The resultant Chern values for the low energy bands are represented for each Δ value with color representation similar to the valence and conduction band. The Berry curvature did not show singularities behavior for the $\Delta = 0$ meV, with resulting $C_{\mathrm{VB/CB}} = 0$. However, for the non zero Δ, the Berry curvatures have singularities at high symmetry points at $\tilde{\mathrm{K}}$ and $\tilde{\Gamma}$ for the conduction band, and at $\tilde{\mathrm{K}}'$ and $\tilde{\Gamma}$ for valence bands, the weight of the singularities varied with applied Δ values. On the right side, the surface plot for the entire mBZ is represented for each case of Δ for the valence and conduction bands. The colorbars on the right represents Ω in arbitrary units.

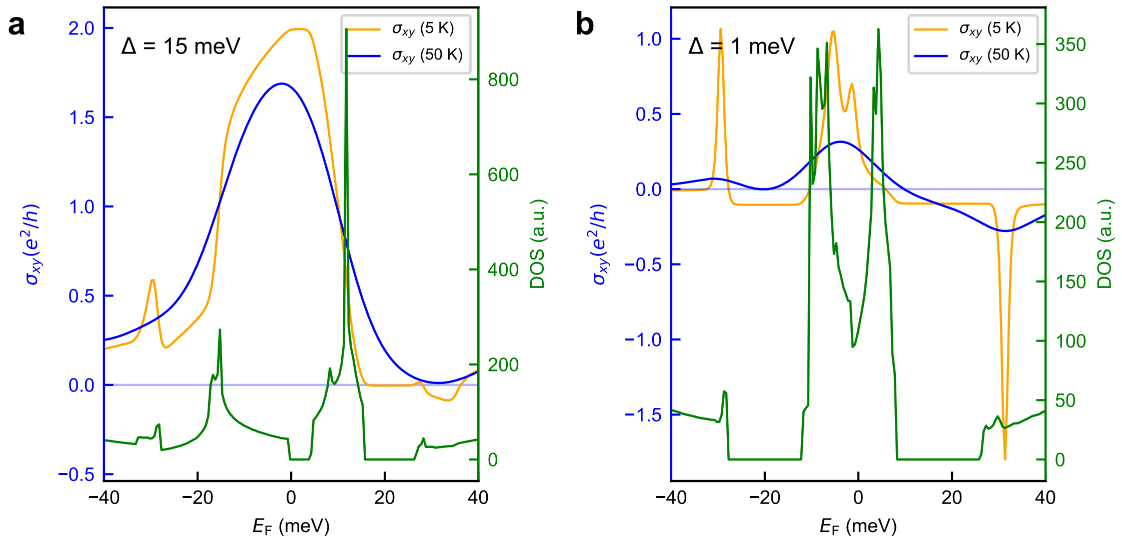

Supplementary Figure 3: Hall conductivity at the CNP. The variation of Hall conductivity (σ_{xy}) (on left axis) for the 1.18° TDBG device as a function of Fermi energy (E_F) at $T = 5$ K (orange curve) and $T = 50$ K (blue curve) for interlayer potential difference $\Delta = 15$ meV (a) and $\Delta = 1$ meV (b). The corresponding variation of density of states (green curve corresponding to the right axis) is overlaid. In (a), the two peaks in the DOS, towards the positive and negative sides of E_F, correspond to the two flat bands separated by a band gap close to $E_F = 0$. In (b), the DOS goes to 0 at the moiré gaps.

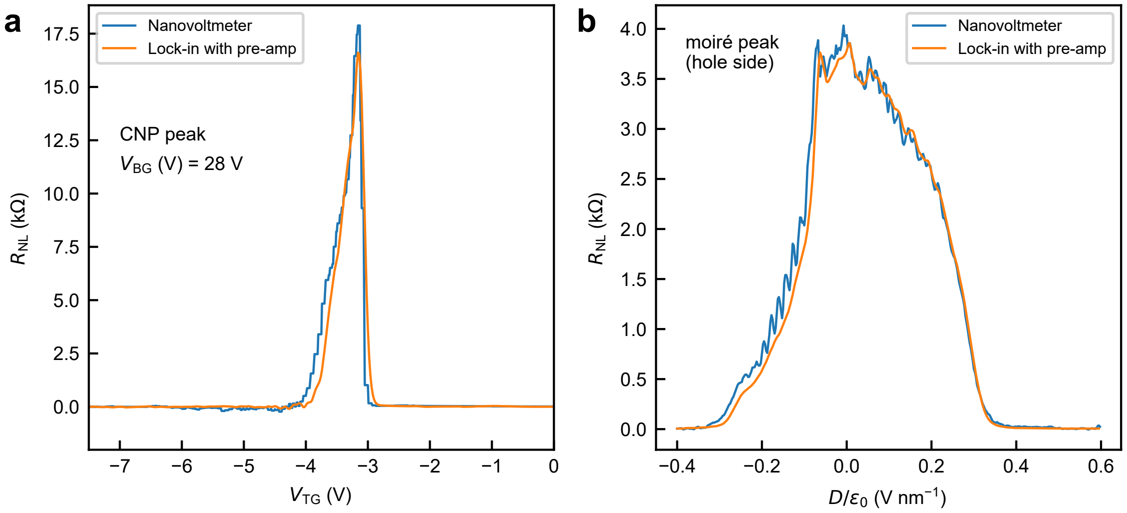

Supplementary Figure 4: Nonlocal resistance measurement using different setups. (a) Nonlocal resistance as a function of V_{TG} at CNP peak for two different measurement schemes – dc measurement using Keithley 2182 nanovoltmeter along with Keithley 6221 current source and ac measurement using lock-in technique with a preamplifier. (b) Nonlocal resistance as a function of the electric field for $n = -n_S$ using the two methods.

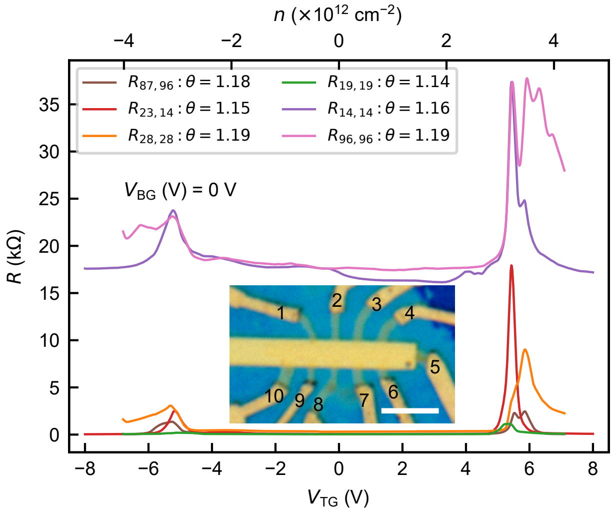

Supplementary Figure 5: Estimating angle inhomogeneity. Variation of two-probe and four-probe local resistance using different terminals of the device as a function of V_{TG} at $V_{BG} = 0$ V. In $R_{ij,kl}$, the indices i, j denote the voltage probes and k, l denote the current probes. The difference between the positions of two moiré peaks is used to estimate the local twist angle. Inset: An optical microscope image of the device with the terminals numbered. The scale bar corresponds to 5 μm.

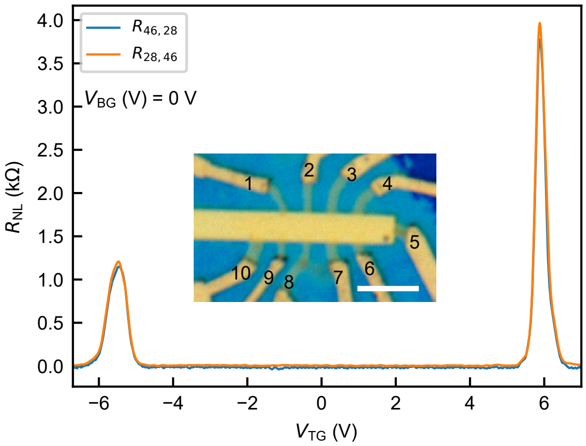

Supplementary Figure 6: Verification of reciprocity of the nonlocal resistance measurement. Nonlocal resistance as a function of V_{TG} with $V_{BG} = 0$ V for two reciprocal combinations of injection and detection probes. In $R_{ij,kl}$, the indices i, j denote the voltage probes (detection terminals) and k, l denote the current probes (injection terminals). The inset shows the image of the device with the scale bar corresponding to 5 µm.

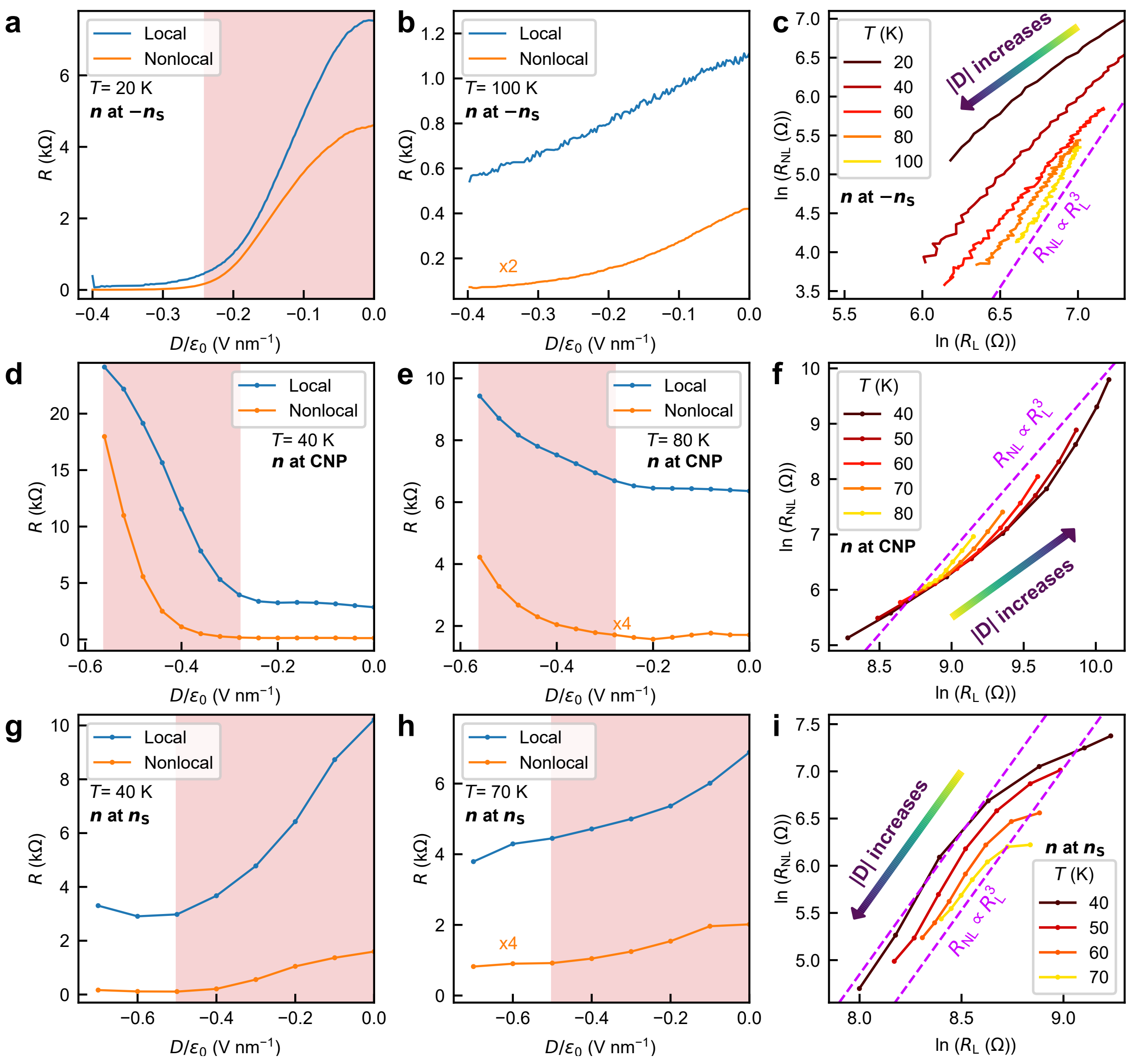

Supplementary Figure 7: Scaling of nonlocal resistance with local resistance at fixed temperatures by taking electric field as parameter. (a-b,d-e,g-h) The variation of nonlocal (orange) and local (blue) resistance as a function of the perpendicular electric field for doping at $-n_S$ (a-b), $n = 0.04 \times 10^{12}$ cm^{-2} (CNP) (d-e), and n_S (g-h). The variation is plotted for fixed temperatures that are indicated in each plot. The nonlocal resistances in (b,e,h) are plotted after multiplying by constant factors that are indicated in orange. The pink background indicates the electric field range used to show scaling in (c,f,i). (c,f,i) The scaling of nonlocal resistance with local resistance with the electric field as the parameter. The scaling is shown for doping at $-n_S$ (c), $n = 0.04 \times 10^{12}$ cm^{-2} (CNP) (f), and n_S (i) for various fixed temperatures. The dashed violet line is a guide to the eye for cubic scaling. The arrows indicate the direction of the rise in the magnitude of the electric field.

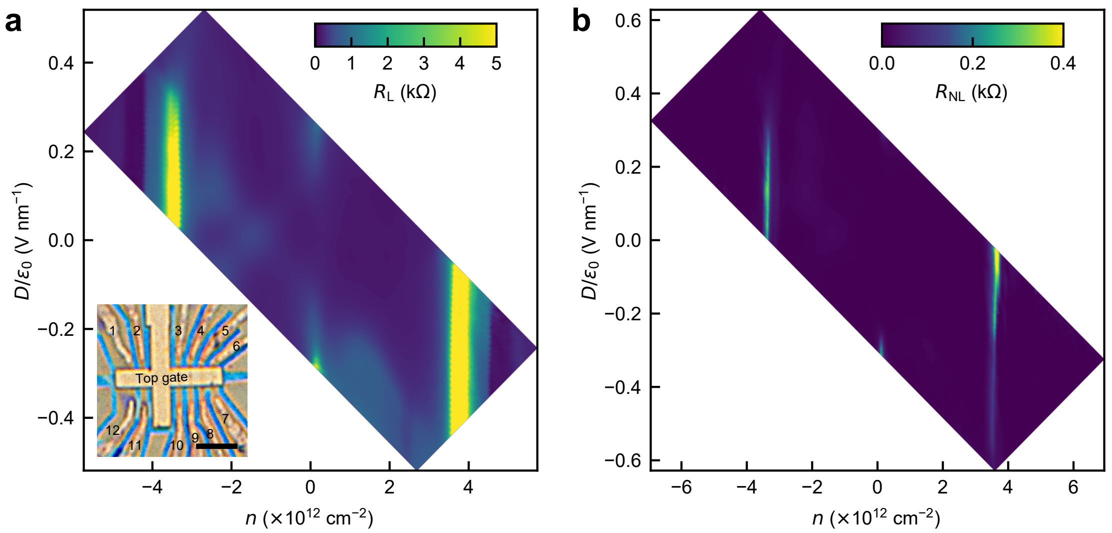

Supplementary Figure 8: Nonlocal resistance measured in Device 2 with twist angle 1.24° at 1.5 K. (a) Color scale plot of local resistance as a function of charge density and electric displacement field. Inset: Optical microscope image of the device with electrodes labeled by numbers. Scale bar corresponds to 4 µm. (b) Color scale plot of nonlocal resistance as a function of charge density and electric displacement field. For measuring the nonlocal resistance, the current was sent through the terminals 9 and 4, and voltage was measured across the terminals 11 and 2.

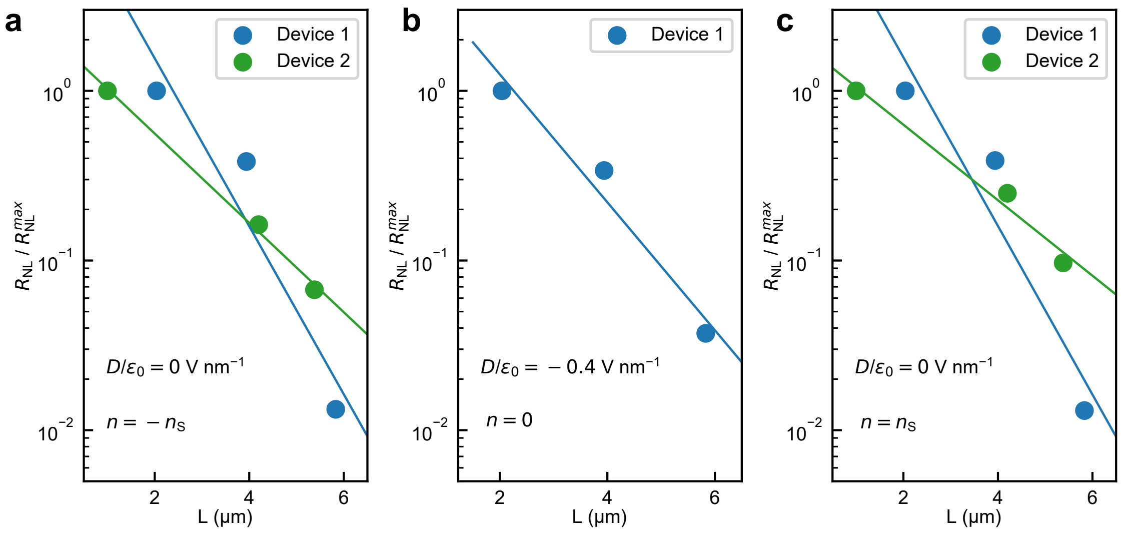

Supplementary Figure 9: Decay of nonlocal resistance over length. (a-c) The variation of the nonlocal resistance as a function of length for $n = -n_S$ (a), $n = 0$ (b) and $n = n_S$ (c) for Device 1 and Device 2. Here, R_{NL} has been normalized with respect to the nonlocal resistance measured in the nearest probe, R_{NL}^{max}, for both the devices.

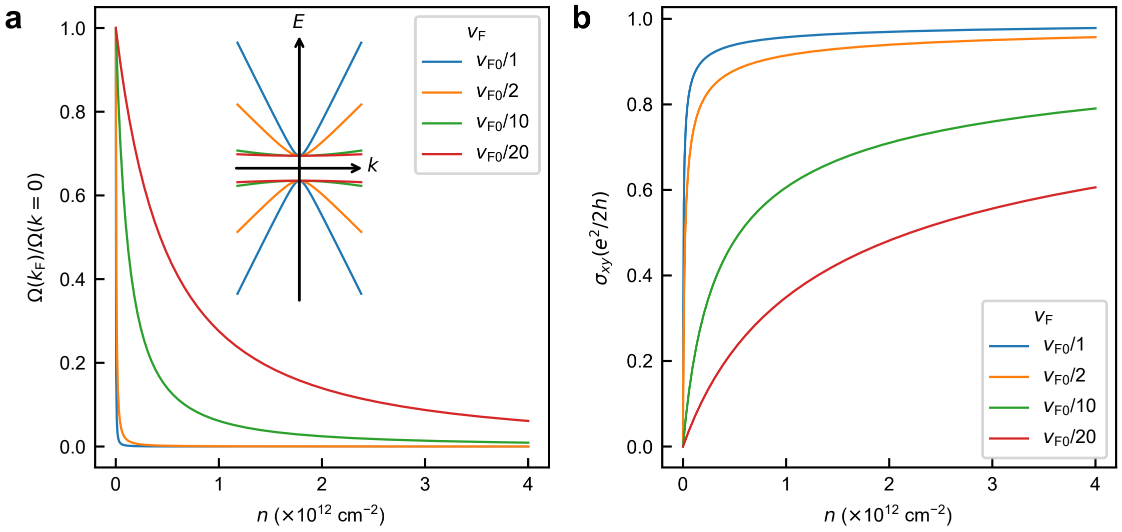

Supplementary Figure 10: Effect of the flat band on Berry curvature hotspot. (a) The variation of the Berry curvature at the Fermi wave vector $\mathbf{k}_\mathrm{F}$ as a function of charge density in the gapped monolayer graphene band for different Fermi velocities. The band gap used for the calculation is 10 meV. The inset shows the band dispersion. (b) The dependence of valley Hall conductivity as a function of charge density for different Fermi velocities. The band gap is 10 meV.

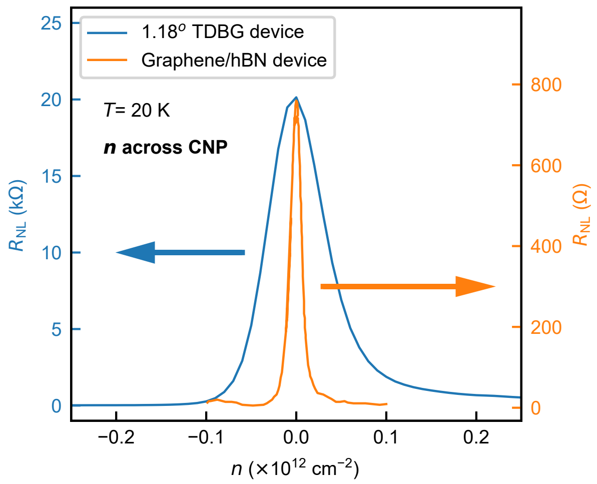

Supplementary Figure 11: Effect of the flat band on the extent of nonlocal resistance peak. The variation of the nonlocal resistance as a function of charge density varying across the charge neutrality point. The blue curve represents the measured nonlocal resistance at the CNP gap at a perpendicular electric field of -0.4 V nm^{-1} in our 1.18° TDBG device at $T = 20$ K. The orange curve is the nonlocal resistance variation across the charge neutrality point from hBN aligned graphene device, which does not have any flat band. The data points in the orange curve are extracted from Gorbachev et al.[1], corresponding to $T = 20$ K.

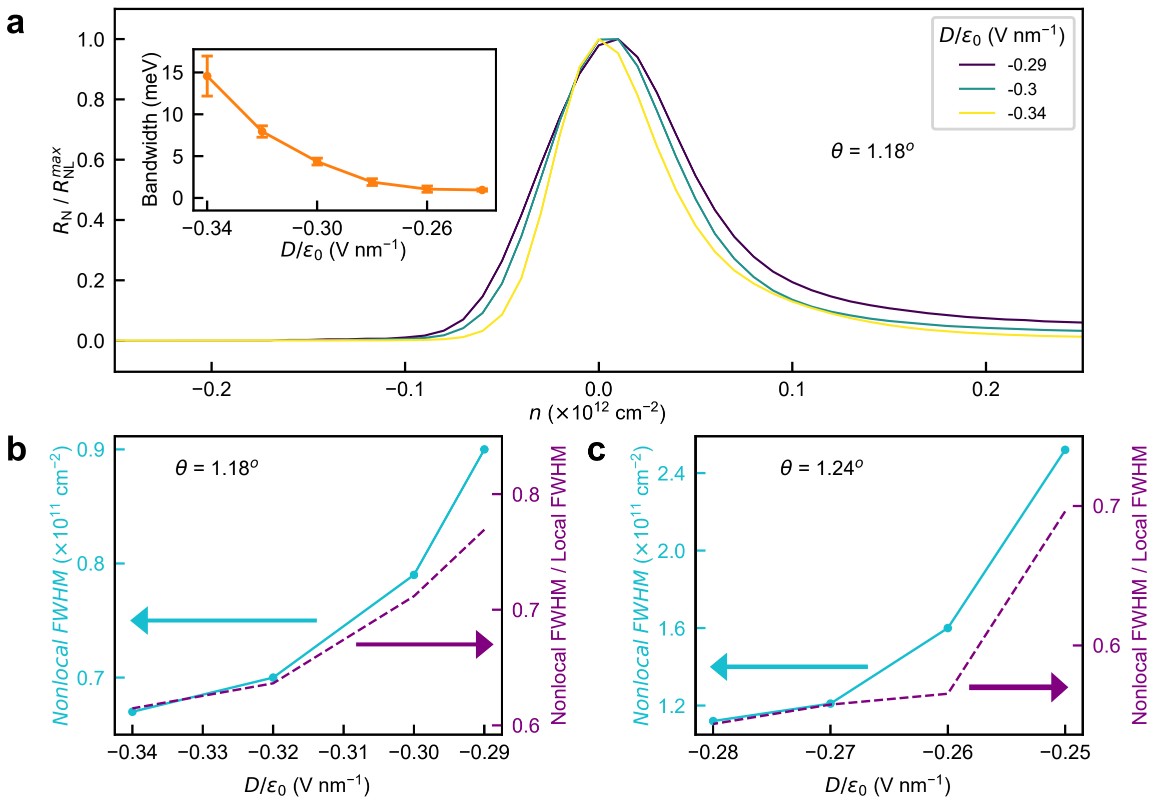

Supplementary Figure 12: Broadening of nonlocal resistance peak in TDBG due to flat bands. (a) Variation of nonlocal resistance (R_{NL}) with charge density (n) in 1.18° TBDG at 9.5 K. The three line slices are plotted after normalizing with respect to the individual maximum resistance at the peak (R_{NL}^{peak}) to compare the FWHM. Inset: Variation of the bandwidth of the flat band in the same D range[2]. Variation of the FWHM of the nonlocal resistance peak (cyan colored curve corresponding to left axis) with electric field at CNP in TBDG with a twist angle of 1.18° (b) and 1.24° (c). The purple curves (right axis) are plotted after normalizing the FWHM of nonlocal resistance peak with that of local resistance at CNP. The data points in (b) are extracted at $T = 9.5$ K (three of the nonlocal resistance curves have been shown in (a)). The data points in (c) are extracted from the 1.24° TDBG device shown in Supplementary Fig. 8.

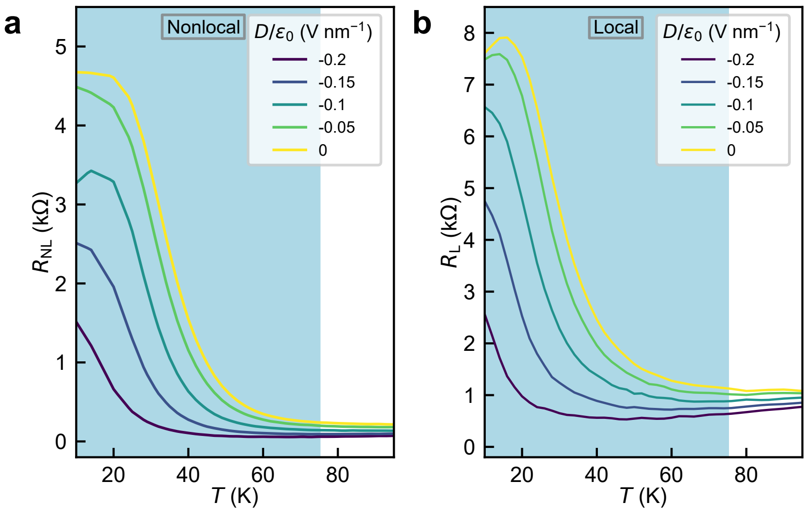

Supplementary Figure 13: Variation of resistance with tempertaure at $n = -n_S$. Variation of nonlocal resistance (a) and local resistance (b) with temperature for the hole-side moiré gap at $n = -n_S$ for the 1.18° TDBG device. The blue-shaded background represents the temperature range used to show scaling in Fig. 3e of main manuscript.

Supplementary Note 1: Calculation of band structure and valley Hall conductivity in TDBG

As discussed in the theory paper Ref.[3], moiré bands theory for the moiré pattern superlattice[4] and the accurate continuum models[5] are used to obtain the electronic structure of the twisted double bilayer graphene (TDBG). The continuum model of Bistritzer-MacDonald for the twisted bilayer graphene (TBG)[4] is extended to the case of twisted double bilayer graphene (TDBG), the Hamiltonian of TDBG at the valley K with the interlayer coupling between the twisted layers through a first-harmonic stacking-dependent interlayer tunneling function, and subject to Δ_i intralayer potentials as

$$H_{\mathrm{TDBG}}(\theta) = \begin{pmatrix} h_{\mathrm{t}}^{+} + \bar{\Delta}_1 & t_s^{+} & 0 & 0 \\ t_s^{+\dagger} & h_{\mathrm{b}}^{+} + \bar{\Delta}_2 & T(\mathbf{r}) & 0 \\ 0 & T^{\dagger}(\mathbf{r}) & h_{\mathrm{t}}^{-} + \bar{\Delta}_3 & t_s^{-} \\ 0 & 0 & t_s^{-\dagger} & h_{\mathrm{b}}^{-} + \bar{\Delta}_4 \end{pmatrix}, \quad (1)$$

where $h_{\mathrm{t/b}}^{\pm} = h_{\mathrm{t/b}}(\pm\theta/2)$ such that the relative twist angle between the bilayers is θ.

The Dirac Hamiltonian given by $h(\theta) = \upsilon_{\mathrm{F}} \hat{R}_{-\theta} \mathbf{p} \cdot \boldsymbol{\sigma}$ includes a phase shift due to a rotation $\hat{R}_{-\theta}$ such that $e^{\pm i\theta_{\mathbf{p}}} \rightarrow e^{\pm i(\theta_{\mathbf{p}}-\theta)}$, where $\boldsymbol{\sigma} = (\sigma_x, \sigma_y)$ and σ_z are the graphene sublattice pseudospin Pauli matrices, and the momentum is defined in the xy plane $\mathbf{p} = (p_x, p_y)$, where we assume K valley unless stated otherwise. The Fermi velocity $\upsilon_{\mathrm{F}} = \upsilon_0$ defined from $\upsilon_i = \sqrt{3}|t_i|a/2\hbar$ is related to the intralayer nearest-neighbor hopping term t_0 = -3.1 eV that captures the experimental moiré band features better[6].

The top and bottom bilayer graphene (BG) are labeled through the positive/negative (+/-) rotation signs, while in turn we have top/bottom (t/b) graphene layers within each BG that are coupled through the matrices $t_s^{\pm}$. The interlayer coupling model of a bilayer graphene is given by

$$t_{\mathrm{AB}}^{\pm} = \begin{pmatrix} -\upsilon_4 \pi^{\pm\dagger} & -\upsilon_3 \pi^{\pm} \\ t_1 & -\upsilon_4 \pi^{\pm\dagger} \end{pmatrix}, \quad t_{\mathrm{BA}}^{\pm} = t_{\mathrm{AB}}^{\pm\dagger}, \quad (2)$$

satisfying $t_{s=+} = t_{s=-}^{\dagger}$ for AB or BA ($s = \pm 1$) stacking-dependent interlayer coupling that consists of a minimal coupling term $t_s = t_1(\sigma_x - is\sigma_y)/2$ plus remote hopping contributions through the terms t_3 = 0.283, t_4 = 0.138 eV, giving rise to trigonal warping and electron-hole asymmetry. The $\pi^{\pm}$ operators include the phases due to $\pm\theta/2$ layer rotation. The Hamiltonian of graphene is given by $h_l^{\pm}(\theta) = h \pm (\theta) + \delta(\mathbb{1} - ls\sigma_z)/2$ where the second term adds a δ =0.015 eV sublattice potential at the higher energy dimer sites at

the t/b layers $l = \pm$[7], that depends on AB or BA stacking $s = \pm$, respectively. The site potentials $\bar{\Delta}_i$ are mapped on its sublattices through $\bar{\Delta}_i = \Delta_i \mathbb{1}$ where i = 1, 2, 3, 4 are the layer labels from top to bottom, and $\mathbb{1}$ is a 2 × 2 identity matrix. Here, we have an additional control knob to change the electronic structure through a perpendicular external electric field that modifies the interlayer potential Δ_i values in equation (1). The potential drops introduced by an external electric field could be modeled through the parameter set $\Delta_1 = -\Delta_4$, $\Delta_2 = -\Delta_3$, redefined as $\Delta_1 = 3\Delta/2$ and $\Delta_2 = \Delta/2$, where Δ is the interlayer potential difference between each BG.

We can identify the interlayer tunneling with the first harmonic expansion coefficient of the interlayer coupling such that $t_1 = 3\omega$[5,7], and for simplicity we use the same AB stacking tunneling within each Bernal BG and the twisted interfaces. In the small-angle approximation, the interlayer coupling Hamiltonian is given by

$$T(\mathbf{r}) = \sum_{j=0,\pm} e^{-i\mathbf{Q}_j \cdot \mathbf{r}} T^j_{l,l'}. \tag{3}$$

where the three $\mathbf{Q}_j$ vectors $\mathbf{Q}_0 = K\theta(0,-1)$ and $\mathbf{Q}_\pm = K\theta(\pm\sqrt{3}/2, 1/2)$ are proportional to twist angle θ and $K = 4\pi/3a$ is the Brillouin zone corner length of graphene, whose lattice constant is $a = 2.46$ Å, and here the indices l, l' label the sublattices of neighboring twisted surface layers. The interlayer coupling matrices between the two rotated adjacent layers are given by

$$T^0 = \begin{pmatrix} \omega' & \omega \\ \omega & \omega' \end{pmatrix}, \quad T^\pm = \begin{pmatrix} \omega' & \omega e^{\mp i2\pi/3} \\ \omega e^{\pm i2\pi/3} & \omega' \end{pmatrix}, \tag{4}$$

using a form that distinguishes interlayer tunneling matrix elements $\omega = \omega_{BA'}$ and $\omega' = \omega_{AA'}$ for different and same sublattice sites between the layers. The convention taken here for the T^j matrices[5] assumes an initial AA stacking configuration $\tau = (0,0)$ and differs by a phase factor with respect to the initial AB stacking $\tau = (0, a/\sqrt{3})$[8,9]. The greater interlayer separation c compared to the carbon-carbon distances a_{CC} lead to slowly varying interlayer tunneling function $T(\mathbf{r})$ and the moiré patterns can often be accurately described within a first-harmonic approximation[4,5]. Furthermore, the effects of atomic relaxation in the moiré patterns lead to corrugations that have non-negligible effects in the details of the electronic structure for both intralayer potentials and interlayer coupling[10]. In the case of $\omega' \neq \omega$, we proposed a single parameter relation through $\omega' = C_1\omega^2 + C_2\omega + C_3$, where $C_1 = -0.5506$, $C_2 = 1.036$, $C_3 = -0.02245$ as discussed in

Ref.[3] (in its Supplementary information). Our calculations have used a configuration space with variable cutoff in momentum space of a radius of up to $6G_1 = 24\pi\theta/(\sqrt{3}a)$ using Hamiltonian matrices with sizes as large as 676×676 such that $\theta \gtrsim \omega/(12\pi|t_0|)$ to obtain converged results in the limit of small θ and large ω.

The possibility of band gap opening at charge neutrality point (CNP), primary gap (δ_p), through an electric field together with the presence of moiré gaps (secondary gaps, δ_s) with the higher-energy bands leads to well-defined valley Chern numbers. The valley Chern numbers were calculated through

$$C_\upsilon = \int_{\text{mBZ}} d^2\mathbf{k}\ \Omega_q(\mathbf{k})/2\pi, \tag{5}$$

by integrating the moiré Brillouin zone for each valley the Berry curvature for the q-th band through[11]

$$\Omega_q(\mathbf{k}) = -2\sum_{q' \neq q} \text{Im}\left[\frac{\langle u_q|\frac{\partial H}{\partial k_x}|u_{q'}\rangle\langle u_{q'}|\frac{\partial H}{\partial k_y}|u_q\rangle}{(E_{q'} - E_q)^2}\right], \tag{6}$$

where for every $\mathbf{k}$ point we take sums through all the neighboring q' bands, the $|u_q\rangle$ are the moiré superlattice Bloch states, and E_q are the eigenvalues.

The Hall conductivity that results due to a nonzero Berry curvature (Ω) at a particular valley of graphene (K or K$'$) is given by[12]

$$\sigma_{xy}(E_\text{F}) = \frac{1}{2\pi}\frac{e^2}{h}\sum_q \int d^2\mathbf{k}\ \Omega_q(\mathbf{k}) f(\varepsilon_q(\mathbf{k}), E_\text{F}), \tag{7}$$

where q indicates the band index and $f(\varepsilon_q(\mathbf{k}), E_\text{F}) = \frac{1}{e^{(\varepsilon_q(\mathbf{k})-E_\text{F})/K_\text{B}T}+1}$ denotes the Fermi occupation function. At low temperature (shown in orange curve for $T = 5$ K in Supplementary Fig. 3), it saturates to $\frac{2e^2}{h}$ in the band gap between the flat bands (Supplementary Fig. 3a). Away from the CNP gap, it starts decreasing since the Berry curvature in the low energy conduction band and valence band have opposite signs, as seen from the line plots in Supplementary Fig. 2. This decrease of σ_{xy} away from the CNP gap in TDBG is asymmetric in nature, which is in contrary to that obtained for bilayer graphene[13,14]. The Hall conductivity is also shown for an elevated temperature of $T = 50$ K (blue curve) where we have observed bulk valley transport at the CNP gap. The decrease in σ_{xy} from its low temperature value of $\frac{2e^2}{h}$ at the gap is due to the thermal excitation of valence band electrons to the conduction band. The valley Hall conductivity, σ_{xy}^{VH}, is obtained by adding the contribution to the Hall conductivity from the individual valleys at K and K$'$, and is given by $\sigma_{xy}^{\text{VH}} = 2\sigma_{xy}$[13].

Supplementary Fig. 3b shows finite non-zero σ_{xy} at the moiré gaps at low interlayer potential of $\Delta =$ 1 meV. Our observation of nonlocal resistance at the moiré gaps is consistent with the fact that nonlocal resistance was also observed at the moiré gaps of other systems like hBN aligned monolayer graphene[1].

Supplementary Note 2: Nonlocal transport measurement

The transport measurement reported in the main manuscript and all other sections in the supplementary was measured using a low frequency ($\sim$ 17 Hz) lock-in technique by sending a current $\sim$ 10 nA and measuring the voltage after amplifying using SR560 preamplifier or preamplifier model 1021 by DL instruments, Ithaca. Since the nonlocal resistance is appreciable when the system is gapped, spurious signal can be measured for these high resistivity states[13,15]. To verify the nonlocal resistance we measured is not a measurement artifact, we employ Keithley 2182 nanovoltmeter to measure dc voltage while sending current using Keithley 6221 current source. The nanovoltmeter has input impedance > 10 GΩ while the SR560 or DL 1021 preamplifier has an input impedance of 100 MΩ. In Supplementary Fig. 4a, we have plotted the nonlocal resistance as a function of top gate voltage V_{TG} around the CNP at $V_{\mathrm{BG}} = 28$ V measured both by the lock-in method aided by the preamplifier and the dc measurement using nanovoltmeter. For measurement using the nanovoltmeter, we measured resistance both in forward and reverse directions and took the average resistance to nullify any dc voltage drop due to thermo-electric effect at various junctions of the current path inside the cryostat. In Supplementary Fig. 4b, we plotted the nonlocal resistance of the moiré peak at the hole side as a function of the perpendicular electric field using the two schemes. As seen from both Figs. 4(a) and 4(b), the resistance values are independent of the measurement schemes. All the measurements subsequently were done using lock-in with the preamplifier.

Supplementary Note 3: Angle homogeneity for nonlocal detection of valley current

The twist angle homogeneity is an important prerequisite for measuring nonlocal valley transport in twisted graphene devices. This is because the generation of valley current in the injection probes happens when the Fermi energy lies in a Berry curvature hotspot, i.e., the Fermi energy lies near a gap. This requires the charge density to be tuned by the gates to specific values: $n = 0$ (CNP gap) or $n = \pm n_{\mathrm{S}}$ (moiré gap), where $n_{\mathrm{S}} = 8\theta^2/(\sqrt{3}a^2)$ with $a = 0.246$ nm being the lattice constant of graphene. Now for detecting the valley current, the detection probes have the same requirement. Since n_{S} depends on θ, the local twist angle should

be the same in both the pairs of injection and detection probes as well as the valley current path.

To estimate the local twist angle near various probes we measure two-probe and four-probe resistance using different combinations of current and voltage probes. In Supplementary Fig. 5, we present such different plots of local resistance as a function of V_{TG} for $V_{BG} = 0$ V. All the curves have two moiré peaks corresponding to $n = \pm n_S$. The difference in the positions of the two peaks on the n-axis corresponds to $2n_S$, which is used to estimate the local twist angle. We find that the twist angle to vary between 1.14° to 1.19°, establishing that the device has good angle homogeneity.

Supplementary Note 4: Reciprocity of the nonlocal resistance measurement

For the Onsager reciprocal relations to be valid, the nonlocal resistance we measure should be the same if one swaps the injection and the detection terminals[13]. We verify this in Supplementary Fig. 6 where we present nonlocal resistance as a function of V_{TG} with $V_{BG} = 0$ V for two reciprocal combinations of injection and detection probes. We find that the nonlocal resistance does not change if we swap the terminals.

Supplementary Note 5: Additional data on scaling

A tell-tale signature of bulk valley current is cubic scaling between the nonlocal and local resistances. In Fig. 3e and Fig. 3f of the main manuscript, we had demonstrated the cubic scaling by taking temperature as a parameter for some fixed electric fields. In Supplementary Fig. 7, we show the cubic scaling at different fixed temperatures for the CNP (Supplementary Fig. 7f) and moiré gaps (Supplementary Fig. 7c for $n = -n_S$ and Supplementary Fig. 7i for $n = n_S$) by taking the electric field (D) as a parameter. For $n = -n_S$ in Supplementary Fig. 7c, we see cubic scaling towards the high temperature end. The case for $n = n_S$ in Supplementary Fig. 7i shows cubic scaling in the high electric field end, while the scaling deviates from cubic and saturates towards the low electric field end where the band gap at $n = n_S$ is higher[2]. This saturation at high band gap regime is attributed to large valley Hall angle physics[16] and is also seen in earlier studies at the CNP gap of bilayer graphene[13]. In Supplementary Fig. 7f we find that the CNP shows cubic scaling at high temperatures. As the temperature is lowered (brown and black curve in Supplementary Fig. 7f), the scaling deviates from cubic to higher exponents in the high electric field end. This is similar to that in Fig. 3f of the main manuscript, where high D and low T shows the same departure.

Supplementary Note 6: Data from the second device

In Supplementary Fig. 8, we present local and nonlocal resistance from Device 2 which has a twist angle of 1.24°.

Supplementary Note 7: Decay of Nonlocal resistance with length

The dependence of the nonlocal resistance on the length of the current path is governed by the valley diffusion length l_v, as seen from equation (8),

$$R_\mathrm{NL} = \frac{1}{2}\left(\frac{\sigma_{xy}^\mathrm{VH}}{\sigma_{xx}}\right)^2 \frac{W}{\sigma_{xx} l_\mathrm{v}} \exp\left(-\frac{L}{l_\mathrm{v}}\right). \tag{8}$$

Here, σ_{xy}^VH is the valley Hall conductivity. L and W represent the length and width of the Hall bar channel, respectively. To extract l_v we plot the decay of the nonlocal signal along the sample length at the moire gaps and the CNP in Supplementary Fig. 9. We fit the exponential decay using equation (8) and extract out l_v for both the devices. The data for Device 1 is plotted at $T = 35$ K to negate low temperature effects. For moiré gaps, we find l_v to be 0.9 µm and 1.8 µm for Device 1 and Device 2 respectively. At the CNP, l_v is 1.15 µm for Device 1. These values are similar to those obtained in bilayer graphene[13].

Supplementary Note 8: Effect of band flatness on Berry curvature hotspot

To understand the effect of a flat band on the Berry curvature hotspot, we consider a simple band structure of gapped monolayer graphene. We choose the monolayer graphene Hamiltonian since, for a flat band, in any twisted system, there is no reason in general for the linear term in momentum to be zero. The Hamiltonian for the gapped monolayer graphene is given by, $H = \hbar v_\mathrm{F} \boldsymbol{\sigma}.\mathbf{k} + \Delta_\mathrm{g}\sigma_3$. Here $2\Delta_\mathrm{g}$ is the band gap between two energy bands given by $E_\pm = \pm\sqrt{(\hbar v_\mathrm{F}\mathbf{k})^2 + \Delta_\mathrm{g}^2}$. We incorporate the band flatness by renormalizing the term v_F in the Hamiltonian, whereas in the case of monolayer graphene $v_\mathrm{F} = 10^6$ m/s $= v_\mathrm{F0}$. The Berry curvature is given by $\Omega(\mathbf{k}) = (\hbar v_\mathrm{F})^2 \Delta_\mathrm{g}/[2((\hbar v_\mathrm{F}\mathbf{k})^2 + \Delta_\mathrm{g}^2)^{3/2}]$. With the charge density n given by $n = \mathbf{k}_\mathrm{F}^2/\pi$ and $\delta = \Delta_\mathrm{g}/\hbar v_\mathrm{F}$, the Berry curvature at the Fermi wave vector $\mathbf{k}_\mathrm{F}$ is given by $\Omega(\mathbf{k}_\mathrm{F}) = \delta/[2(n\pi + \delta^2)^{3/2}]$. In Supplementary Fig. 10a we plot the Berry curvature as a function of charge density for monolayer graphene with a gap of $2\Delta_\mathrm{g} = 10$ meV and for different values of the renormalized velocity, v_F. As v_F is smaller, i.e., the band is flatter, we find that the Berry curvature is more delocalized away

from the gap. In Supplementary Fig. 10b we plot the contribution of the conduction band to the valley Hall conductivity given by, $\sigma_{xy} = (e^2/2h)\int \Omega(\mathbf{k})kdkd\theta = (e^2/2h)(1-\delta/\sqrt{n\pi+\delta^2})$. We again find that in the case of flat band the Hall conductivity saturates to its asymptotic value much slower. The Berry curvature delocalization and slow saturation of Hall conductivity in flat band systems, as represented in Supplementary Fig. 10, can be reproduced even by starting with a gapped bilayer graphene Hamiltonian.

Supplementary Note 9: Comparison of extent of nonlocal resistance peak with a system without flat bands

Here we compare the extent of the nonlocal signal in the charge density axis from our TDBG device with that of hBN aligned graphene device from Gorbachev et al.[1]. We compare the charge neutrality peak at $T = 20$ K for both the devices. Upon application of a perpendicular electric field (shown for $D/\varepsilon_0 = -0.4$ V nm^{-1} in Supplementary Fig. 11), a gap opens up at charge neutrality within the flat bands in the TDBG device[2]. These bands have Berry curvature hotspots, as shown in Supplementary Fig. 2. For the case of hBN aligned graphene, the superlattice potential results in opening up a gap between the valence band and conduction band at the charge neutrality[17]. This, too, results in Berry curvature hotspots at the band edges near the gap opening. However, unlike the case in small-angle TDBG, the resulting low energy bands are not flat. The broader extent of the nonlocal resistance in our data (the FWHM being ∼5 times larger) can be attributed to the spreading of Berry curvature hotspot due to flat bands in the TDBG system.

Supplementary Note 10: Modulation in FWHM of nonlocal peak by tuning Fermi velocity

We now further demonstrate that the broadening of the nonlocal resistance peak in the charge density axis is indeed due to Berry curvature hotspot spreading as Fermi velocity, v_{F}, decreases. We can tune v_{F} by changing electric field within the same TDBG device having a fixed twist angle. This is because the perpendicular electric field can modulate the bandwidth of the flat bands in TDBG, as shown in the inset of Supplementary Fig. 12a and also discussed in Chebrolu et al.[3]. As the bandwidth reduces, that is the band becomes flatter, the v_{F} reduces. We have plotted the nonlocal resistance in the 1.18° TDBG as a function of charge density across the CNP for three different electric fields in Supplementary Fig. 12a. We find that as we increase the electric field $|D|$ (i.e., as the bandwidth of flat bands increases), the width of the nonlocal resistance peak becomes narrower, thus confirming that lower Fermi velocity at low electric field results in wider nonlocal

resistance peak.

We systematically extract the FWHM and plot its variation with the electric field for two devices with twist angle 1.18° (Supplementary Fig. 12b) and 1.24° (Supplementary Fig. 12c). We find that this feature of increase in FWHM of R_{NL} with decrease in v_{F} of flat bands (by decreasing $|D|$) is repeatable (left axis corresponding to cyan colored curve in Supplementary Fig. 12b and Supplementary Fig. 12c). To further rule out any effect of local resistance, we also plot the FWHM of nonlocal resistance peak after normalizing with the FWHM of local resistance (right axis corresponding to purple colored curve in Supplementary Fig. 12b and Supplementary Fig. 12c), which shows the same trend. An increase in the ratio by decreasing electric field $|D|$ signifies that a decrease in Fermi velocity of the flat bands spreads the Berry curvature hotspots in **k**-space–this translates to expanding the FWHM width of the nonlocal resistance peak at CNP. This reinforces our understanding of the physics of Berry curvature spreading due to flat bands with low v_{F}.

SUPPLEMENTARY REFERENCES

[1] Gorbachev, R. V. *et al.* Detecting topological currents in graphene superlattices. *Science* **346**, 448–451 (2014).

[2] Adak, P. C. *et al.* Tunable bandwidths and gaps in twisted double bilayer graphene on the verge of correlations. *Phys. Rev. B* **101**, 125428 (2020).

[3] Chebrolu, N. R., Chittari, B. L. & Jung, J. Flat bands in twisted double bilayer graphene. *Phys. Rev. B* **99**, 235417 (2019).

[4] Bistritzer, R. & MacDonald, A. H. Moiré bands in twisted double-layer graphene. *Proceedings of the National Academy of Sciences* **108**, 12233–12237 (2011).

[5] Jung, J., Raoux, A., Qiao, Z. & MacDonald, A. H. Ab initio theory of moiré superlattice bands in layered two-dimensional materials. *Phys. Rev. B* **89**, 205414 (2014).

[6] Wong, D. *et al.* Local spectroscopy of moiré-induced electronic structure in gate-tunable twisted bilayer graphene. *Phys. Rev. B* **92**, 155409 (2015).

[7] Jung, J. & MacDonald, A. H. Accurate tight-binding models for the π bands of bilayer graphene. *Phys. Rev. B* **89**, 035405 (2014).

[8] Javvaji, S., Sun, J.-H. & Jung, J. Topological flat bands without magic angles in massive twisted bilayer graphenes. *Phys. Rev. B* **101**, 125411 (2020).

[9] Leconte, N., Jung, J., Lebègue, S. & Gould, T. Moiré-pattern interlayer potentials in van der waals materials in the random-phase approximation. *Phys. Rev. B* **96**, 195431 (2017).

[10] Jung, J., DaSilva, A. M., MacDonald, A. H. & Adam, S. Origin of band gaps in graphene on hexagonal boron nitride. *Nature Communications* **6**, 6308 (2015).

[11] Xiao, D., Chang, M.-C. & Niu, Q. Berry phase effects on electronic properties. *Rev. Mod. Phys.* **82**, 1959–2007 (2010).

[12] Lado, J. L. & Fernández-Rossier, J. Quantum anomalous Hall effect in graphene coupled to skyrmions. *Physical Review B* **92**, 115433 (2015).

[13] Shimazaki, Y. *et al.* Generation and detection of pure valley current by electrically induced Berry curvature in bilayer graphene. *Nature Physics* **11**, 1032–1036 (2015).

[14] Koshino, M. Electronic transport in bilayer graphene. *New Journal of Physics* **11**, 095010 (2009).

[15] Sui, M. *et al.* Gate-tunable topological valley transport in bilayer graphene. *Nature Physics* **11**, 1027–1031 (2015).

[16] Beconcini, M., Taddei, F. & Polini, M. Nonlocal topological valley transport at large valley hall angles. *Phys. Rev. B* **94**, 121408 (2016).

[17] Song, J. C. W., Samutpraphoot, P. & Levitov, L. S. Topological Bloch bands in graphene superlattices. *Proceedings of the National Academy of Sciences* **112**, 10879–10883 (2015).
